# Clinicians as advocates amid refugee resettlement agency closures

**DOI:** 10.1057/s41271-021-00296-9

**Published:** 2021-07-21

**Authors:** Hafifa Siddiq, Julia Rosenberg

**Affiliations:** 1 National Clinician Scholars Program, Division of General Internal Medicine and Health Services Research, University of California, Los Angeles, 1100 Glendon Ave. Suite 900, Los Angeles, CA 90024 USA; 2 Yale University School of Medicine, Yale University, New Haven, CT USA

**Keywords:** Refugees, Clinician advocate, Resettlement agency, Health policy, Health services, Healthcare disparities

## Abstract

As ongoing war and violence forcibly displace people worldwide, resettlement remains a critical response to the unprecedented global refugee crisis. In recent years, however, the USA (US) has diminished admissions, forcing agencies to shutter offices and resettlement programs across the nation—posing a silent threat to the refugee resettlement system. We provide historical context of refugee resettlement, discuss challenges, and offer recommendations for healthcare providers to become more effective advocates for refugee health in the USA. The need is urgent for healthcare providers and institutions—particularly in regions of high resettlement—to advocate for expanding and assuring sustainable capacity to care for refugees. Key elements include promotion of trauma-informed care, integration of social services in primary care settings, partnership with community-based organizations to promote continuation of care, advocacy for resources and services, and opposition to policies detrimental to the health of refugees and immigrants.

## Key messages

1. As the refugee crisis reaches unprecedented magnitude, the USA drastically diminished refugee admissions in recent years—threatening the national refugee resettlement system.
2. Healthcare providers can play a critical role in advocating for refugees by providing trauma-informed care, integrating social services within primary care, developing community partnerships to address social service needs, and promoting continuity of care.
3. Healthcare providers should address structural and social determinants of refugees’ health, advocate improved policies for refugees, and recognize the repercussions of anti-immigration policies on refugees’ health and wellbeing.

## Introduction

Amid a global refugee crisis, the entire United States (US) refugee resettlement system is in peril [1]. As of mid-2020, the number of displaced people worldwide had reached an unprecedented 80 million and the numbers of those displaced is projected to continue to increase [1]. Among the world’s displaced populations, 26.3 million are refugees [1]. Recent anti-immigrant policies during the Trump Administration and the drastic reduction of refugee admissions in the USA may have far-reaching adverse consequences for the health of refugees and the sustainability of the US resettlement program [2]. Resettlement agencies provide critical resettlement services including housing placement and employment assistance for newly resettled refugees in the USA and help refugees start their lives. As a result of diminishing admittance of refugees into the USA since 2017, resettlement agencies have shuttered more than 100 out of an estimated 325 programs and offices across the nation [3, 4]. Systematic decimation of this infrastructure threatens critical refugee health screening and social services programs, leaving refugees and their surrounding communities to deal with the consequences. To again serve the numbers of refugees as it has for decades, the USA must rebuild the infrastructure and human power of the refugee resettlement system. Healthcare providers should play critical roles, addressing health care needs and advocating for resettled refugee communities–and can become a stronger force.

In this Viewpoint, we provide an overview of the now crumbling US refugee resettlement system—crumbling as a result of policies introduced by the Administration of President Trump (2017–2021) [2]. Temporary suspension of refugee resettlement during the COVID-19 pandemic exacerbated deterioration in capacity of the US system [5]. We set the US system in context internationally, then highlight the role of US resettlement agencies to address newly resettled refugees’ social service and health care needs. Resettlement agencies face solvency challenges in a politicized climate that has denigrated refugees. Healthcare providers in outpatient and primary care settings can address an urgent need to advocate for sustainable capacity to care for refugees, particularly in regions of high resettlement. Providers can also promote trauma-informed care, integration of social services in primary care settings, partnership with community-based organizations to foster continuity of care, advocacy for resources and services, and opposition to policies detrimental to the health of refugees and immigrants. Because many aspects of the US refugee resettlement system remain in flux, the policies and systems described below may continue to evolve. See Table 1 for common terms and definitions.

**Table 1:** Terms and Definitions

*US immigration categories*	
Asylee	A person fleeing persecution or serious danger, and granted, by a State, protection on its territory
Asylum seeker	A person fleeing persecution or serious danger and seeking international protection
Displaced population	People who involuntarily leave their homes as a result of a natural, technological or deliberate event. Displaced populations include refugees, asylum seekers and internally displaced people
Internally displaced people (IDP)	People who are displaced within their own countries and remain under the protection of government, even if that government is the reason for their displacement
Immigrant	An individual who voluntarily leaves their country of origin and enters another country to reside permanently
Lawful permanent resident	Lawful permanent residents (LPRs), also known as “green card” holders, are non-citizens who are lawfully authorized to live permanently within the U.S
Refugee	The 1951 Refugee Convention is a key legal document and defines a refugee as: “someone who is unable or unwilling to return to their country of origin owing to a well-founded fear of being persecuted for reasons of race, religion, nationality, membership of a particular social group, or political opinion.”
*Organizations and roles*	
Case manager or social worker	A staff person at a resettlement agency assigned to assist a refugee in navigating social service programs. In general, the role of the case manager is to undertake assessment, monitoring, planning, advocacy and linking people with rehabilitation and support services
Ethnic-community-based organization	A community-based organization, comprised primarily of refugees, for the specific purpose of providing assistance to other refugees
Health navigator	An individual or organization trained and able to help consumers, small businesses and their employees as they look for health coverage options through the ‘Marketplace’, including completing eligibility and enrollment forms
Office of Refugee Resettlement	A program within the US Department of Health and Human Services that provides resources for refugees, asylum seekers, and other new arrivals to the US to assist with their integration into their new community
Refugee resettlement program	A program responsible for coordinating and funding some refugee-specific services and benefits, including RMA
Refugee resettlement system	A public–private partnership that assists in the permanent resettlement of refugees
Resettlement agency	A non-profit organization that has a cooperative agreement with the federal government to assist refugees in resettling in the United States
United Nations High Commissioner for Refugees	A global agency mandated to aid and protect refugees, to assist in their voluntary repatriation, local integration, or resettlement to a third country
United Nations Relief and Works Agency	A global agency, established by the United Nations General Assembly of 1949, to carry out direct relief. works programs specifically for Palestine refugees
United States (US) Citizenship and Immigration Services	An agency of the United States Department of Homeland Security that administers the country's naturalization and immigration system
US Immigration and Customs Enforcement	A federal law enforcement agency under the US Department of Homeland Security that enforces immigration and customs laws
*US services and programs*	
Medicaid	A federal health care program run by the state that assists low-income families or individuals in paying for doctor visits, hospital stays, and long-term medical care
Children’s Health Insurance Program (CHIP)	A federal health care program for that provides low-cost health insurance to children in families that earn too much money to qualify for Medicaid
Refugee Medical Assistance (RMA)	A temporary medical benefit funded by the federal government available to refugees and other eligible beneficiaries
Refugee resettlement	The transfer of refugees from the country in which they have sought asylum to another that has agreed to admit them. The refugees will usually be granted asylum or some other form of long-term resident rights and, in many cases, will have the opportunity to become naturalized citizens
Social service programs	Programs administered by the federal, state, or local government using government funding directed at reducing poverty, improving opportunities for low-income adults or children, promoting self-sufficiency, rehabilitation, and include services like Medicaid, SNAP, and WIC
Supplemental Nutrition Assistance Program (SNAP)	A federal program that provides food-purchasing assistance for low- and no-income people
Women, Infant, & Children (WIC)	A federal program that provides breastfeeding support, nutritious foods, information on healthy eating, and referrals to health care to pregnant, postpartum and breastfeeding women and children up to age five

## Context for US refugee resettlement: United Nations and refugees around the world

After the atrocities of World War II, the international community held the 1951 Refugee Convention in Geneva to adopt protocols and outline a framework for legal protections and social rights for protecting refugees [6]. At that time, the United Nations (UN) established the UN Health Commissioner for Refugees (UNHCR)—a global agency mandated to aid and protect refugees, to assist in voluntary repatriation, and local integration, or facilitate resettlement to a third country, or both. According to the 1951 Refugee Convention, a refugee is defined as a “person who is outside his or her country of nationality or habitual residence; has a well-founded fear of being persecuted because of his or her race, religion, nationality, membership of a particular social group or political opinion; and is unable or unwilling to avail him- or herself of the protection of that country, or to return there, for fear of persecution” [6]. Refugees are forced to flee due to a threat of persecution and lack protection of their own country. An immigrant, in comparison, may leave his or her country for other reasons like employment, family reunification, or study, and may do so under the protection of their country of origin [6]. (See Table 1 for definitions of US immigration categories.)

Resettlement is a vital response to an ongoing global refugee crisis. Resettlement offers a pathway to safety, freedom, and independence and a humane alternative to refugee camps [2]. According to the UNHCR, there are over 20 million refugees worldwide but only less than one percent of them are resettled each year [7]. Globally, countries resettled 92,000 refugees in 2018, down from 103,000 in 2017 and a peak of 189,000 in 2016 [8, 9]. The USA has historically resettled more refugees annually than all the other resettlement countries in the world combined. After the USA that resettled 33,000 refugees in 2017, Canada resettled 27,000 refugees and Australia resettled 15,000 refugees. In 2017, Canada led other resettlement countries in resettlement per capita by resettling 725 refugees per 1 million residents, followed by Australia (618), and Norway (528). By contrast, the USA resettled only 102 refugees per 1 million residents in 2019 [9]. As resettlement in the USA dramatically decreased refugee admissions in recent years, the rest of the world followed suit. In 2018, only 25 countries committed to resettling refugees, down from 37 in 2016 [10]. This decrease in numbers of people resettled across the world and the decrease in countries’ commitment to refugee resettlement is occurring when the number of refugees is increasing each year. Projected worsening of conflicts in fragile states and concurrent climate change will only add to the dramatic rise in displaced populations, increasing the severity and urgency of the refugee crisis [11, 12].

## The US refugee resettlement system

The US refugee resettlement system, a public–private partnership that assists in the permanent resettlement of refugees within the US, is the most extensive in the world—a distinction held by the country since the passage of the Refugee Act of 1980. This Act solidified the relationship between the public (made up of governmental agencies) and private organizations (nongovernmental organizations). It established a national refugee resettlement program (comprised of services provided by resettlement agencies and refugee-serving organizations) to help refugees integrate and achieve self-sufficiency in the US [13]. The Act, established after the atrocities of World War II and passed with bipartisan support, aligns with the United Nation’s 1948 Universal Declaration of Human Rights that guarantees the right of refugees to seek protection in other countries [13].

Since the passage of the Act, the USA has resettled more than 3 million refugees [1]. Each year, the US President initiates the resettlement process by setting an annual limit for the number of refugees the USA will accept. Refugees undergo intensive screening that involves multiple federal departments and overseas processing of each applicant by the US Citizenship and Immigration Services [14]. Former President Trump set an historic low number of refugees to be admitted each year he was in office, setting the refugee ceiling at 18,000 in 2020 and 15,000 refugees in 2021—a more than sevenfold reduction from 110,000 refugees allowed in 2017 [15]. In addition, from March through June 2020, the UNHCR suspended resettlement travel for refugees because of the COVID-19 pandemic [16]. Despite promising to reverse Trump era limits on refugee admissions, President Biden upheld the refugee admission cap of 15,000, a number set by former President Trump, sparking resistance from human rights groups [17]. As a result, President Biden announced in 2021 the revision of the US annual admissions to cap to 62,500 and stated “This erases historically low number set by the previous administration of 15,000, which did not reflect America’s values as a nation that welcomes and supports refugees” [17].

The US refugee resettlement systems involve coordination of services between federal agencies, nongovernmental organizations (NGOs), and local services organizations [18]. The US Refugee Act of 1980 established the Office of Refugee Resettlement (ORR), responsible for providing resources for refugees, asylum seekers, and other new arrivals to the USA to assist them with integration to become self-sufficient [2]. The ORR allocates funds based on the number of refugees resettled in each state; funding is dependent on the arrival of refugees [19]. When refugees arrive in the USA, their sponsoring refugee agency receives a Reception and Placement grant of $2,175 per refugee to pay for certain expenses, including food, rent, and job placement services during the initial 90 days of resettlement [4, 18, 19]. Other funding sources include longer-term state grants or private donations to help refugees beyond the initial 90 day resettlement period. Case managers who work with refugee clients typically prepare, rent, and furnish an apartment for an incoming refugee family. Most often, the case manager uses these funds before the refugee arrives in the USA.

## Refugee health care provisions

The US Department of Health and Human Services requires refugees to undergo mandatory medical exams pre-departure and upon arrival in the USA. Medical facilities provide primary health care services in the USA, not refugee agencies. Upon arrival, refugees become eligible for Refugee Medical Assistance (RMA), a form of short-term health insurance, for up to eight months. After this period, refugees must obtain public or private health care insurance to pay for care [20, 21]. The RMA covers similar medical expenses as Medicaid (a federal and state program that helps with healthcare costs for people with limited income and resources) and includes coverage for a required domestic medical examination within 90 days of arrival in the USA. Some refugees may be eligible for government health insurance such as Medicaid for their first 7 years in the country [20].

Gaps in health insurance coverage remain for refugees and other immigrants in the USA. Based on an analysis of 2018 American Community Survey data, non-citizens are substantially more likely to be uninsured compared to US citizens [22]. Among non-citizens, about 23% of lawfully present immigrants (including refugees, asylees, and other lawful permanent residents) lack health insurance to pay for medical services [22]. These gaps in coverage demonstrate a need for policymakers to consider promoting the continuation of health insurance to cover health services especially when RMA coverage lapses after eight months.

## Refugee health and barriers to health care

Refugees experience a triple burden of ill health: non-communicable diseases, infectious diseases, and mental health disorders [23]. Before resettlement, refugees may experience violence, trauma, and exposure to endemic communicable diseases. After resettlement, these exposures and their effects add to the stresses of integrating into a new society and increase the risk for developing chronic health problems endemic to high-income countries of resettlement [24–26]. Robust population-level surveys among refugees are scarce, and reports of the prevalence of mental health disorders vary widely, from 20 to 80 percent among refugees [27]. These may include anxiety, post-traumatic stress disorder (PTSD), depression, and somatization. According to the American Psychiatric Association, approximately 1 in 3 refugees and asylum seekers appear to suffer from at least one of these mental health conditions [28]. Refugees are at particular risk for mental health challenges because civilians in war zones typically experience at least one traumatic event due to war, and war refugees often are subjected to torture, sexual violence, and other traumatic events [29].

Healthcare providers play a critical role in promoting resilience and mitigating adverse effects of trauma for immigrant and refugee families through ‘trauma-informed care’. Trauma-informed care refers to the context in which trauma is addressed among people receiving services and how caregivers incorporate fundamental trauma principles in organizational culture. Principles of trauma-informed care include promoting safety, building trusting relationships, providing peer support, collaborating with patients and families, promoting a sense of control and empowerment, and acknowledging cultural, historical and gender issues [30]. The American Academy of Pediatrics recommends that caregivers use a trauma-informed care approach with immigrant and refugee families [31].

Post-migration stressors such as economic hardship and social and cultural challenges of adjustment to the USA compound the burden on refugees already experiencing trauma and other risk factors for mental illness [30, 32]. Despite being more likely to work than their US-born counterparts, refugees are also more likely to have lower income than the rest of the general population [33]. Evidence shows how poor socioeconomic status and disadvantaged living conditions are associated with worse mental health [32]. In addition, criminalization of immigrants and experiences like discrimination and racism and sentiment against immigrants generally harm health and mental health [34, 35].

Although refugees are eligible for health insurance in the USA, they underutilize services, including mental health care, dental care, and preventive health services including vaccination and cancer screening services, compared with their US-born counterparts [36, 37]. Refugees and those who provide health and social services to refugees report that a significant barrier to the use of health services is limited English language proficiency and lack of knowledge about health resources and available services [30]. Other barriers include lack of provision of health service information upon arrival in the USA, limited understanding of the complex healthcare and specialty services in the USA, and logistical issues related to lack of transportation or availability of interpreters [30]. Stigma toward mental health and unfamiliarity with preventive health care may also further inhibit refugees’ use of services [34].

## Role of refugee resettlement agencies

Refugee resettlement agencies promote integration of refugees and help them to set up their lives in the USA. These agencies provide resettlement services including housing placement and employment assistance to promote self-sufficiency as soon as possible. The agencies also provide newly resettled refugees with vital resources for social services, and especially important, interpretation services [38]. Because the USA admits refugees from over 150 countries, culturally and specific language- and dialect-tailored services are critical–and even more so as refugee populations are increasing in diversity.

To mitigate unfamiliarity with the complex array of social and health services and overcome barriers to care, refugee resettlement agencies provide case management, coordinating services for people with complex needs, and collaborate with health services [39]. Resettlement agencies may be staffed by case managers, social workers, health navigators, or other support staff with roles that may overlap with one another when working with refugee clients. For example, case managers may conduct social needs screening for refugees and other clients related to social support, food insecurity, social isolation, housing safety, homelessness, transportation barriers, job placement, and financial insecurity. They also assist refugees to sign up for social service programs, and may provide peer support in the home. So called ‘health navigators’ may conduct health-related screening, provide nutrition and physical health literacy, assist in enrolling in health care coverage, schedule required medical exams, and secure transportation or language interpreters when available.

Agency staff can promote communication between designated refugee clinic staff and the resettlement agency and frequently work closely with nearby healthcare providers, using their familiarity with supplemental resources and services–such as nutrition and health services listed in Table 1 [38]. Answering the call to integrate social care into health care delivery—a call championed by organizations like the National Academies of Sciences, Engineering, and Medicine—may help bring together resources within the primary care setting to support refugee patients [38].

Refugee resettlement agencies may also connect refugees with local ethnic community-based organizations (E-CBOs) or other community networks including faith-based organizations or social groups that serve key roles in the integration of refugees within communities. E-CBOs are community-based organizations comprised primarily of refugees that assist other refugees. Services offered through E-CBOs may overlap with resettlement agencies and provide counseling, medical care, youth development, employment counseling, social adjustment services, cultural preservation and information, and referral services while continuing to address the unique cultural needs of that community. They often have culturally and linguistically competent staff to help connect knowledgeable community members with recently arrived refugees. When refugees no longer receive resettlement agency services during their initial resettlement period, E-CBOs attempt to fill refugees’ needs for longer-term support and services for the community without regard to duration of stay in the USA [18, 38].

## Socio-political climate and challenges for refugee resettlement agencies

Policies and executive orders by the Trump administration may have negatively impacted the overall health and wellbeing of refugee populations in the USA [40]. The anti-immigrant Trump era socio-political climate contributed to anti-immigrant policies, including the lowered cap of refugee admissions, detention of a large proportion of asylum seekers in US Immigration and Customs Enforcement (ICE) facilities, tightened restrictions on immigration and travel bans, and further restricted public charge rules (described below) [40–44].

The reduction in refugee admissions, coupled with suspensions of refugee resettlement programs related to the COVID-19 pandemic, threatens the future of refugee resettlement in the USA [4]. Inconsistent numbers of refugees admitted undermine federal funding. In turn, the funding cuts threaten US protection of refugees because refugee resettlement agencies rely on federal funding allocated per refugee sponsored. Dwindling admissions have driven staff layoffs and office closures [18]. Over 100 of 325 agency offices and organizations, including some that had been providing essential services to the community for half a century, have reportedly closed their doors, and many more may be in precarious situations amid the COVID-19 pandemic [4]. In addition, an executive order in 2017 banned arrival or re-entry of refugees and other migrants from Muslim-majority countries of Syria, Iraq, Somalia, Yemen, Iran, Sudan, and Libya, an order then reversed by current President Biden [42]. The American Civil Liberties Union documented the devastating impact on US families through family separations and uprooting of lives, not only at the border, but also overseas where the US government barred family reunification [45]. The dominant populations in countries included in this ban were made up of individuals of color (non-white). These countries also have substantial Muslim populations.

The former Trump Administration attempted to reduce the number of people eligible for permanent residency with tightening of ‘public charge rules’ [43]. These rules permit government to deny immigrants visas or entry into the US due to disabilities or lack of economic resources. Such policy changes create a chilling effect to decrease enrollment in federal benefit programs, even for immigrants who have legal entitlement to the benefits [44]. The programs offer social and health services include: Supplemental Nutritional Assistance Program (SNAP), Women, Infants and Children (WIC), Medicaid for children, and publicly subsidized housing. SNAP and WIC provide food-purchasing assistance for low- and no-income people. Confusion about which rules apply to whom and about use of which programs may adversely affect immigration status—often leaves eligible refugees uncertain about what services they may use legally. Researchers estimated in 2019 that one in seven adults in immigrant families avoided social service programs due to concerns about public charge rules [43]. Thus, thousands of eligible, low-income children do not benefit from legally available government support, even during the severe COVID-19 health and economic crisis [44].

## Recommendations for healthcare providers to support refugees

The burdens to refugees’ wellbeing that we enumerate above amount to ‘structural determinants’ of ill health [46, 47]. Healthcare providers should be aware of root causes of health inequities experienced by refugees and advocate for improvements in the US refugee resettlement program. These root causes are ‘structural’ and include economic, social, and political mechanisms that cause health and social inequalities [46]. Providers can advocate against discriminatory policies targeting immigrants and refugees and recognize the consequences to health of structural barriers, such as poverty and racism. These barriers inhibit attaining a living wage through decent jobs, quality education, and secure housing and safe communities. In addition to supporting changes in national policy under the Biden Administration (starting in 2021) to reverse the problems caused by the Trump administration, we offer recommendations for how healthcare providers can contribute to meeting the comprehensive health and social needs of refugees by leveraging existing community-based resources.

### Recommendations

1. **Promote trauma-informed care**. Healthcare providers can promote resilience and mitigate negative impacts of pre-migration trauma among refugee youth and families with trauma-informed care [30, 48, 49]. Healthcare providers can make clinical encounters therapeutic while minimizing re-traumatization, if they:
  Establish a relationship with the patient prior to referring clients to local and trusted resources and ask permission before discussing traumatic events or performing invasive procedures.
  Inform patients that they may request a providers or interpreters or both by certain attributes, such as gender. Many refugees are not aware of this right.
  Assure that screening and follow-up for mental and physical health issues are sensitive to patients’ cultural beliefs and values even when these do not align with Western biomedical models of treatment.
  Promote a safe space for immigrants and refugees in healthcare settings and involve administrators, supervisors, and other ancillary staff who will be crucial for successful adoption and implementation of trauma-informed care.
2. **Integrate social and mental health services into primary care**. Healthcare providers can work with their practice teams and use community resources to integrate social and mental health services within the healthcare setting with the following [50–53]:
  Ensure that integrated care service providers have and use support services to learn about specfic cultural needs of newly resettled refugees.
  Screen for and address social service needs routinely.
  Provide language interpreters to improve quality and availability of care, to ensure clear communication and to help providers learn refugees’ cultural conceptions of health and treatment and refugees’ ways of expressing mental health symptoms.
  Engage community resources and clinic support staff to address unmet social service needs and limitations in primary care during the COVID-19 pandemic.
  Use ‘tele-health’ to expand social and mental health services for patients with limited transportation or other barriers, and ensure interpretation for all virtual services.
  Educate and inform refugee patients about confidentiality of in person and tele-health visits.
3. **Promote Partnerships with Community-Based Organizations**. Healthcare providers can work with the local community to address the complex needs of refugees in the following ways [43]:
  Identify refugee resettlement trends and culturally specific and culturally sensitive community resources in the local region.
  Include case managers and health navigators to promote continuity of care and ensure timely health screenings and follow-up.
  Refer patients to local culturally tailored community mental health services when needed.
  Promote collaboration and clear communication between healthcare providers and community organizations to improve responses to one another’s needs through timely and coordinated efforts.
  Foster a partnership between clinics with refugee resettlement agencies and other NGOs working with immigrant and refugee communities, including those that provide trauma-informed social and legal services.
  Strengthen community-academic partnerships by seeking opportunities to share resources and funding.
4. **Educate and advocate for support and funding for services to support refugees.** Healthcare providers should recognize detrimental effects of anti-immigration policies and advocate for refugees’ health and wellbeing by [54]:
  Educate nurse, physician and other healthcare professionals-in-training about immigrant health, including the structural barriers and social determinants of health.
  Encourage these trainees to use social media and other means to contact local representatives of the city or state, to express their views on policies and amplify the effects of advocacy.
  Combine health professionals’ voices with individual and organizational-level initiatives to advocate for legislative support and funding to improve health of immigrants and refugees through.
5. **Familiarize Oneself with and Harness Key Resources and Tools for Providers.**
  Use an online tool for service providers that allows each provider to help their clients understand if use of public health benefits may interfere with clients’ options for gaining their desired immigration status [55].
  Educate trainees to make use of tools (such as the US Centers for Disease Control and Prevention (CDC) and EthnoMed), that provide refugee health profiles for major refugee groups in the USA [56, 57].
  Engage with refugee support networks and become involved with advocacy, research, and practice groups, including the Society of Refugee Healthcare Providers and the American Public Health Association Caucus of Immigrant and Refugee Health to make use of these tools [58].
  Use an online toolkit to implement changes in policy and action that promote the physical and psychological safety of immigrant patients in hospital and clinic settings [59].

## Conclusion

The atrophy of the US resettlement system has far-reaching sequelae for the present and future of the US refugee resettlement program and its intended beneficiaries. Trump era policy changes stoked anti-immigrant sentiment and destabilized the resettlement agency system. The need to promote a sustainable refugee resettlement system in the USA is urgent. Health professionals can increase capacity and improve care for immigrant and refugee families by using trauma-informed care, integrating social and mental health services in primary care settings, partnering with refugee resettlement agencies to promote continuation of care, and advocating for policies and legislation to fund these initiatives.
